# Effects of *Weizmannia faecalis* DSM 32016 and *Bacillus licheniformis* DSM 33806–Based Probiotics on Performance, Carcass Traits, and Intestinal Health of Broilers

**DOI:** 10.3390/ani16071010

**Published:** 2026-03-25

**Authors:** Vassilios Dotas, Panagiotis Sakkas, Ilias Giannenas, Despoina Karatosidi, Lydia Zeibich, Alexandra Schlagheck, Dimitrios Verros, Nikolaos Lykos, Dimitrios Koutsianos, Marina Gaitanidou, Georgios Theodorou, Eleni Dalaka, George K. Symeon

**Affiliations:** 1 School of Agriculture, Faculty of Agriculture Forestry and Natural Environment, Aristotle University of Thessaloniki, 54636 Thessaloniki, Greece; vdotas@agro.auth.gr (V.D.); gcmarina@agro.auth.gr (M.G.); 2 School of Veterinary Medicine, Faculty of Health Sciences, Aristotle University of Thessaloniki, 54636 Thessaloniki, Greece; psakk@vet.auth.gr (P.S.); igiannenas@vet.auth.gr (I.G.); 3 Research Institute of Animal Science, ELGO-DIMITRA, 58100 Giannitsa, Greece; despinakaratosidi@elgo.gr; 4 Biochem Zusatzstoffe Handels- und Produktionsgesellschaft mbH, 49393 Lohne, Germany; zeibich@biochem.net (L.Z.); schlagheck@biochem.net (A.S.); verros@biochem.net (D.V.); lykos@biochem.net (N.L.); 5 Vet Analysis, 12462 Chaidari, Greece; dkoutsianos@yahoo.gr; 6 Department of Animal Science and Aquaculture, Agricultural University of Athens, 11855 Athens, Greece; gtheod@aua.gr (G.T.); elenidalaka@aua.gr (E.D.)

**Keywords:** probiotics, *Weizmannia faecalis*, *Bacillus licheniformis*, performance, carcass composition, intestinal health

## Abstract

Probiotics have emerged as an important strategy to achieve improved feed efficiency and carcass quality. To evaluate the effects of a probiotic combination based on *Weizmannia faecalis* (formerly *Bacillus coagulans*) and *Bacillus licheniformis* on broiler performance, carcass, and intestinal health, a study was conducted. As-hatched ROSS 308 broilers were purchased from a local hatchery at day 0 and were randomly allocated to two treatments (160 birds per treatment; 8 replicates of 20 birds each): the control, which was fed a standard commercial diet throughout the experiment, and the probiotics group, where the standard diet was further supplemented with the probiotic combination. Feed and water were offered for ad libitum consumption while the feeding schedule was as follows: Starter, 1–10 days, mash; Grower, 11–24 days, mash; Finisher, 25–42, mash. The birds were challenged using re-used litter as bedding and the application of increased stocking density and mild heat stress. The final body weight of the probiotics’ supplemented group was higher than the control at 42 days of age (2822.7 g vs. 2575.4 g, respectively) (*p* < 0.05), and the overall feed conversion ratio was significantly reduced. The weight of all the commercial parts increased, along with the thigh and drumstick yield, thus indicating an improvement of carcass composition (*p* < 0.05). The European Poultry Efficiency Factor significantly improved following the probiotic dietary supplementation (409.7 vs. 344.9 of the control), while the probiotic fed birds had higher antibody titers for Bursal disease at 42 days and lower serum concentration of fatty acid binding protein 2 at 24 days (*p* < 0.05). Overall, the dietary supplementation of broilers with the probiotic mixture, under challenging rearing conditions, enhanced growth performance and improved carcass composition.

## Simple Summary

Modern poultry production is about converting growth performance into commercial value through improved feed efficiency and carcass quality. This can be achieved only with the use of specific feed additives and probiotics have emerged as one of the most promising alternatives to antibiotics in broiler nutrition. After conducting an experiment where the diets of broilers have been supplemented with a mixture of two probiotics, the results showed that they can enhance growth performance and improve carcass composition, even under challenging rearing conditions. Moreover, the probiotics supported the immune system of broilers and protected the intestine from epithelial injury. Therefore, according to these results, their efficacy is confirmed and when their use is coupled with precision nutrition approaches, their adoption by the poultry industry will certainly increase in the future.

## 1. Introduction

Achieving optimal growth in broilers is not only about reaching higher final weights, but also about converting that growth into commercial value through improved feed efficiency and carcass quality. Historically, such performance gains in poultry production were strongly supported by antibiotic growth promoters (AGPs), which improved gut health, growth rate, and ultimately carcass development [1]. After their EU ban in 2006, due to concerns about antimicrobial resistance and consumer expectations, therapeutic antibiotic use has remained common only to manage disease, highlighting the ongoing need for effective non-pharmaceutical solutions [2].

Probiotics are defined by the WHO as “live microorganisms which when administered in adequate amounts confer a health benefit on the host” [3] and have emerged as one of the most promising AGP alternatives in broiler nutrition and as an important preventive strategy to reduce antibiotic interventions [4]. The term microorganisms refers to bacteria (mainly belonging to the genera *Bacillus*, *Lactobacillus*, *Enterococcus*, and *Bifidobacterium*), but certain yeasts and fungal probiotics have also been used, e.g., *Aspergillus oryzae*, *Candida pintolopesii*, *Saccharomyces* and *Saccharomyces cerevisiae*.

Evidence indicates that probiotics can change the microbial population dynamics in the gastrointestinal tract by enhancing the multiplication of beneficial microbes and suppressing the harmful ones [5]. Moreover, they increase the digestion and absorption of nutrients [6] and prevent the chronic inflammation of the gastrointestinal tract through stimulation of innate immunity in the epithelium [7], overall resulting in enhanced performance and feed conversion.

*Weizmannia faecalis* DSM 32,016 (formerly classified as *Bacillus coagulans*) is a Gram-positive, facultative aerobic, and spore-forming bacterium with unique properties. It is a homofermentative lactic acid producer [8,9], performs very well in harsh environments (high temperatures and low pH) [10], and produces compounds with antimicrobial activity against Gram-positive and Gram-negative food pathogens [11]. These properties contribute to its wide use in supporting gut and overall health in both humans and broilers, as well as other farm animals [12,13].

Similarly, *Bacillus licheniformis* is a spore-forming, Gram-positive, facultatively aerobic bacterium that exerts heat and pH resistance and produces bacteriocins that mainly fight Gram-positive pathogens. It has been proven to enhance broilers’ growth performance [14] and to maintain gut microbial population balance [15]. It can also assist in preventing the occurrence of necrotic enteritis in chickens [16] and has been recognized by the European Food Safety Authority (EFSA) as safe for animal dietary use.

Considering the proven efficacy of dietary supplementation with the probiotic microorganisms *Weizmannia faecalis* DSM 32,016 and *Bacillus licheniformis* DSM 33,806 under non-challenged conditions [17], the aim of the present study was to evaluate the effects of this probiotic combination on broiler performance, carcass quality, and intestinal health under the challenges of increased stocking density, reused litter, and mild heat stress, with a particular focus on carcass optimization.

## 2. Materials and Methods

As-hatched ROSS 308 broilers (*n* = 320) were purchased from a local hatchery and were brought to the experimental facilities of the Research Institute of Animal Science in Paralimni Giannitson, which comprise solid floored pens (100 × 100 cm) featuring chopped wheat straw bedding. Upon arrival, the chicks were individually weighed and randomly allocated to two treatments, each consisting of 8 replicates of 20 birds each (160 birds per treatment). The experimental treatments consisted of the control group, which was fed a standard commercial diet throughout the experiment (C), and the probiotics group, where the standard diet was further supplemented with *Weizmannia faecalis* DSM 32,016 and *Bacillus licheniformis* DSM 33,806 (Technocare, Biochem, Lohne, Germany) at a rate of 200 g/t (Pr), according to product specifications. Feed and water were offered in bell feeders and nipple drinkers, respectively, for ad libitum consumption. The birds were provided with a mash diet across three phases: Starter (days 1–10), Grower (days 11–24), and Finisher (days 25–42). The composition and calculated analysis of experimental diets are presented in Table 1. To determine total tract nutrient digestibility (TTD), titanium dioxide was incorporated into the diets at a concentration of 5 g/kg to serve as an indigestible marker.

The lighting regimen consisted of continuous illumination (24 h) for the first three days, followed by a 23L:1D cycle until the end of the third week, and 20L:4D for the remainder of the study. Ambient temperature gradually decreased from 32 °C on day 0 to 19 °C on day 42 (except from the hours when heat stress was applied). The chicks were vaccinated at the hatchery for infectious bronchitis and Newcastle disease, and a second application of the vaccines was performed at 14 days of age. No other supplement was given to the birds throughout the experiment.

To evaluate the efficacy of the probiotic dietary supplementation in challenged birds, three challenges were applied during the experiment: used litter from a previous experiment was used as bedding, elevated stocking density was applied (>39 kg/m^2^ from day 30 and onwards, above the upper limit of stocking density permitted by EU regulations), and mild heat stress (28 °C, for 3 h at midday, days 28–38, 25–35% above suggested room temperature according to the supplier’s management handbook [18]).

The duration of the experiment was 42 days. On day 42, 24 birds per group, sex-balanced (3 per pen), were slaughtered for the recording of carcass measurements. The birds were fastened 12 h before slaughter. They were sacrificed via decapitation and allowed to exsanguinate for approximately 1 min. Carcasses were then scalded (60 °C for 2 min), mechanically plucked (2 min), and manually eviscerated. Following processing, carcasses were chilled at 4 °C for 24 h.

Body weight (BW) and feed intake (FI) were recorded on a per-pen basis at the conclusion of each feeding phase (days 10, 24, and 42). Mortality was monitored daily throughout the experimental period. Flock uniformity was assessed by calculating the coefficient of variation (CV) of body weights within each pen. These primary data were used to determine average daily gain (ADG), feed conversion ratio (FCR), and the European Poultry Efficiency Factor (EPEF).

For the measurement of total tract digestibility, excreta were collected per pen at the end of each feeding phase (days 10, 24 and 42). Excreta samples were subsequently analyzed for dry matter (DM), crude protein (CP), fat content (FC) and fiber content (FiC) using the routine procedures [19]. The total tract digestibility of nutrients was calculated using the following equation:

TTD = 1 − [(Td/Tf) × (Nf/Nd)] × 100

where N and T are the nutrient and titanium concentrations, respectively, while the markers f or d represent the subsequent quantity in the feces and diet, respectively (% DM).

At slaughter, all carcasses were scored for footpad lesions, according to a five-point scale developed by Riber et al. [20]. After slaughter (24 h), the weights of cold carcass, thigh, drumstick, liver, abdominal fat and carcass yield (on the basis of cold carcass to live BW) were also recorded. The weight and length of the tibiotarsus bone were also recorded and the Seedor index (weight/length) calculated [21].

At the ages of 10, 24 and 42 days, blood samples were collected from 3 birds per pen (24 per group) in 15 mL falcon tubes without coagulant, after slaughter. The samples were centrifuged at 3200× *g* for 1 h, and the collected serum was stored at −80 °C until the determination antibodies titers and FABP2 concentration. Serum antibody titers for Infectious Bursal Disease (IBD), Infectious Bronchitis Virus (IBV), and Newcastle Disease Virus (NDV) were determined using commercial ELISA kits (CK113, CK119, and CK116, respectively; BioChek BV, Reeuwijk, The Netherlands) following the manufacturer’s instructions. Briefly, serum samples (1:500 dilution) were processed and read at 405 nm. Antibody status was defined by Sample-to-Positive (S/P) ratios, with positivity thresholds set at ≥0.35 for NDV and ≥0.20 for IBD and IBV. Antibody titers were calculated using the following kit-specific equations:

NDV: Log_10_ Titer = 1.0 × Log_10_(S/P) + 3.52

IBV: Log_10_ Titer = 1.0 × Log_10_(S/P) + 3.62

IBD: Log_10_ Titer = 1.1 × Log_10_(S/P) + 3.361 Serum FABP2 (fatty acid binding protein 2) concentrations were measured using a commercial sandwich ELISA kit specific to chicken FABP2 (SEA559Ga; Cloud-Clone Corp., Katy, TX, USA). The ELISA detection range was 0.78–50 ng/mL, and the LLD was <0.29 ng/mL, as reported by the manufacturer. Serum samples were thawed on ice, gently mixed, and assayed in duplicate without dilution. The ELISA procedure was performed exactly as described by the manufacturer: 100 μL of standards, blank, and samples were added to the pre-coated wells and incubated for 1 h at 37 °C, followed by incubation with Detection Reagent A (1 h at 37 °C), washing, incubation with Detection Reagent B (30 min at 37 °C), further washing, and color development with TMB substrate for 10–20 min. The reaction was stopped with 50 μL stop solution, and absorbance was measured at 450 nm.

Performance data (BW, FI, antibody titers, and FABP2) were analyzed via two-way ANOVA with group, age, and their interaction as fixed effects. Carcass traits, bone properties, and footpad scores were analyzed via one-way ANOVA with group as the fixed effect. The pen served as the experimental unit. Data was screened for outliers based on plausibility and verified for normality and homoscedasticity. Results are expressed as least square means ± pooled SE. Mean comparisons were performed using the Student–Newman–Keuls (SNK) test, with significance defined at *p* < 0.05. All analyses were conducted using STATGRAPHICS (v18, Windows).

## 3. Results

### 3.1. Growth Parameters and Total Tract Digestibility of Nutrients

Concerning the body weights, no significant differences were recorded for days 0, 10 and 24, while at the end of the experiment (D42), the final body weight of probiotic-supplemented broilers was higher than the control group (*p* < 0.05) (Table 2). The average daily gain was comparable between the treatments for the starting and middle period of growth, while for the third phase (D25–42), the probiotic group had the highest ADG (*p* < 0.05). Feed intake was not different among groups at any stage of growth (*p* > 0.05). On the other hand, FCR was lower at D42 in the probiotic group (*p* < 0.05), indicating that the probiotic supplementation significantly improved feed conversion. Regarding the uniformity of the flocks, the coefficient variation (CV) of the body weights per feeding period was not different between the experimental groups, and it ranged between 8 and 10% (Table 2).

In the starter period, the probiotic supplementation improved the digestibility of all three nutrients (*p* < 0.05) (Table 3). In the growing period, the differences persisted for fat and fiber content, while the digestibility of crude protein tended to be higher in the probiotics group (*p* < 0.10). In the finisher period, no statistical differences were recorded between the two groups (*p* > 0.05) (Table 3).

### 3.2. Carcass Parameters, Bone Properties and EPEF

**Figure 1:**
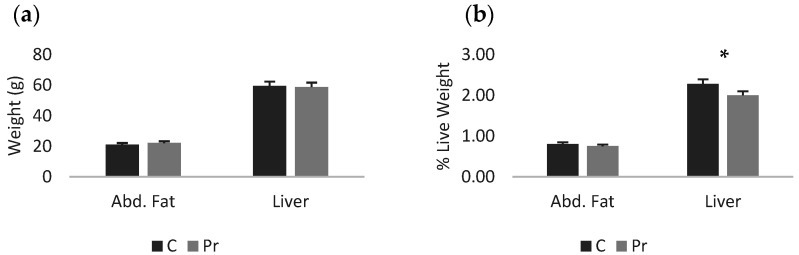
Abdominal fat and liver of the experimental groups presented as absolute weights (**a**) and proportions of live weight (**b**) (* = *p* < 0.05).

**Figure 2:**
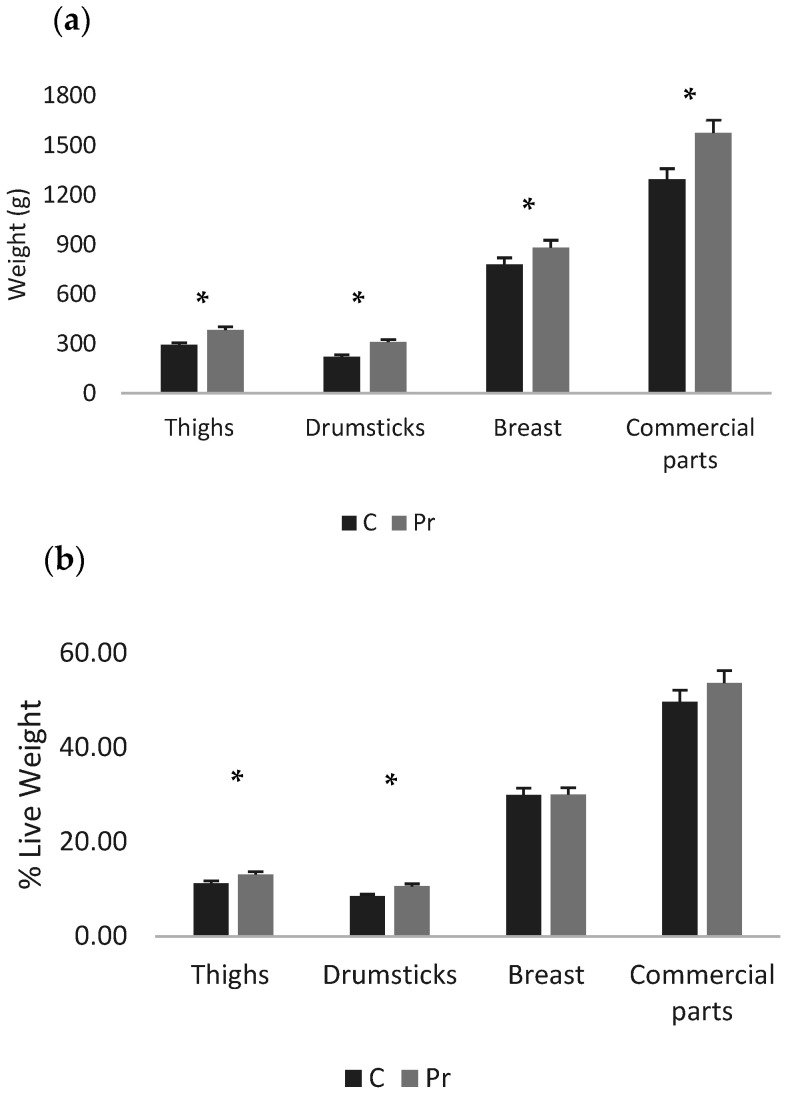
Carcass parts of the experimental groups presented as absolute weights (**a**) and proportions of live weight (**b**) (* = *p* < 0.05).

The probiotics group had higher live and cold carcass weight, while carcass yield was not different between groups (Table 4). In terms of absolute weights of the abdominal fat and liver, there was no statistically significant difference between the experimental groups, while in terms of proportions, the probiotics group had a significantly lighter liver than the control (Figure 1a,b). Moreover, regarding the absolute weights of the main carcass parts, the probiotics group had heavier thigh, drumstick, breast and the commercial parts (two feet plus the breast (*p* < 0.05) (Figure 2a)). In fact, the probiotic supplementation increased thigh weight by 31.1%, drumstick weight by 40.3%, breast weight by 13.1% and commercial parts weight by 21.8%. In proportion to the live weight, significant differences were recorded only for the thigh and drumstick (Figure 2b). Nevertheless, the relevant increases were 16.4% and 24.5% for the thigh and drumstick, respectively, as well as 0.3% for the breast and 8.1% for the commercial parts.

Tibiotarsus weight and the Seedor index were not different among groups, but the probiotics group had a higher average length of that bone (*p* < 0.05) (Table 4). Footpad scoring, on the other hand, was comparable for both groups at the two ages examined. In terms of EPEF, the index that considers body weight gain, feed conversion and mortality (1.7 and 1.2 for the control and probiotics group, respectively), it was significantly higher in the probiotics group, indicating a positive effect of the probiotic supplementation on the overall performance of broilers (Table 4).

### 3.3. Antibodies Titer and FABP2 Concentrations

For the first two diseases, no differences were recorded between the experimental groups, at any age (Table 5). For IBD, while the antibody titer was comparable between the two groups at 10 and 24 days of age, the probiotics group exhibited a higher antibody titer than the control at 42 days of age (*p* < 0.05) (Table 5).

**Figure 3:**
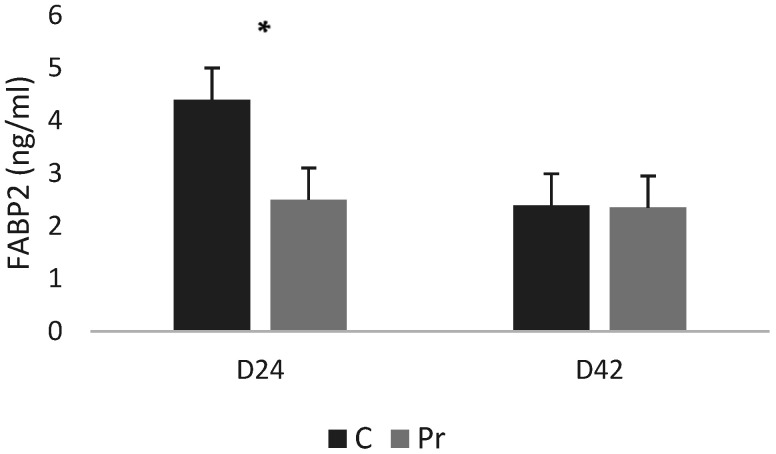
FABP2 (fatty acid binding protein 2) serum concentrations of experimental groups at 24 and 42 days of age (* = *p* < 0.05).

With respect to the FABP2 concentrations, the control group exhibited significantly higher serum FABP2 concentrations (4.4 ng/mL) compared with the birds that were receiving the probiotics (2.5 ng/mL) (Figure 3). Nevertheless, at 42 days, FABP2 concentrations were similar between treatments, with both groups averaging approximately 2.3 ng/mL.

## 4. Discussion

It is well documented that the addition of probiotics in broilers’ diets improves growth performance [22,23], mainly in terms of absolute body weight and weight gain [6,24], an effect that was also observed in this study. With respect to the probiotic microorganisms used in this study, other authors have observed the same positive effects either by using single cultures of *Weizmannia faecalis* (*Bacillus coagulans*) [13,25,26], single cultures of *Bacillus licheniformis* [27,28] or a combination of both probiotics [17]. In terms of feed intake, most researchers agree that it is not affected by the dietary supplementation of probiotics [15,25,29], and, therefore, the increased growth is a result of a better utilization of nutrients by the birds.

The previous assumption is verified by the digestibility results of this study, which have shown that the total tract digestibility of crude protein, fat and fiber are elevated in the birds receiving the combination of probiotics, for the first two feeding phases. Probiotic supplementation significantly improved protein, fat, and fiber digestibility during these early phases, when endogenous enzyme secretion, absorptive capacity, and microbial functionality are still immature and limit nutrient utilization [30]. Although not directly demonstrated in the present study, it can be hypothesized that the probiotic may support the development of a more efficient gastrointestinal tract, potentially enhancing nutrient utilization early in life. This assumption is supported by previous literature reporting that probiotics can promote intestinal morphological development, including increases in villus height and absorptive surface area [31,32]. By the finisher phase, digestive capacity is largely mature and operates close to its physiological maximum, leading to a ceiling effect.

In general, probiotics are widely reported to improve nutrient digestibility in broilers [33,34], mainly through an enhancement of digestive enzyme activities (e.g., trypsin, amylase, protease) [35,36], an improvement of gut morphology [35,37] and the modulation of gut microbiota towards the beneficial bacteria [6]. Specifically, the more pronounced effects have been observed in the improvement of digestibility of dry matter [38], crude protein and selected amino acids [34] and gross energy [39] and less for fat and starch [33,39]. Overall, irrespective of the proposed mechanisms, the combination of increased body weight and unaffected feed intake that was recorded in this study leads to a reduced feed conversion ratio [10,17], an increased EPEF [36,40] and collectively a better economic output of poultry production.

During this study, three challenges were applied to the birds to better evaluate the efficacy of the probiotic dietary supplementation: used litter, elevated stocking density and mild heat stress. The results proved that the challenges were successful, since the final weight of the control group and feed conversion were lower and higher, respectively, to the broiler objectives demonstrated by Aviagen for Ross 308 [18], while the probiotic supplementation helped alleviate these negative effects. Although the present study did not include an unchallenged baseline group, the reduced performance of the control birds compared with breeder objectives indicates that the flock was exposed to moderate production stress. Future studies including both challenged and unchallenged controls would allow a more precise quantification of the challenge severity and the magnitude of probiotic-mediated mitigation. Nunes et al. [41] also found that a combination of probiotic microorganisms (*Lactobacillus acidophilus*, *Enterococcus faecium* and *Bifidubacterium bifidum*) improved the performance of broilers raised on re-used litter. Moreover, it has been reported that dietary supplementation with probiotics can help broilers cope with the adverse effects of high stocking density [42,43,44] or heat stress [45,46,47].

Carcass composition is largely dependent on growth performance and live weight. In this study, the increase in body weight recorded after the dietary supplementation with probiotics had apparent effects on carcass composition, increasing the weight of all the commercial parts. Obviously, if body weight was not affected, the same would apply for the major carcass parts, as it has already been reported elsewhere [48,49]. In terms of the parts’ yield in relation to live weight, there are quite diverse results in the literature. Khan et al. [50] found that the addition of probiotics increased the breast and drumstick yield, Kaushal et al. [51] reposted an increase for breast only with no differences for thigh and drumstick, while Pelicano et al. [52] reported a significant difference only for the thigh. Finally, lower liver yield was also reported by Giang et al. [53]. These diverse results are justified due to the variation in diets, probiotics and management practices used in the related papers.

Probiotics, through the modification of gut microbiota and several physiological pathways, have the ability to influence bone formation and properties in broilers and other animal species [54]. The combination of probiotics used in this study increased tibiotarsus length but did not affect either the weight of the bone or the weight/length index. Javid et al. [55] also reported that a probiotic supplementation increased tibia length, but in their study, bone weight and weight/length index were also higher. On the contrary, Mutus et al. [56] did not find any effect of a probiotic supplementation with *Bacillus licheniformis* and *Bacillus subtilis* on bone properties. Other studies have reported more prominent effects, e.g., an increase in mineral content [57] as well as an increase in the content of Ca and P in the bone [58].

Footpad dermatitis causes necrotic lesions on the footpads of broilers and its prevalence if affected by a lot of factors, with litter moisture being reported as the most prominent factor [59]. Some researchers suggested that the addition of probiotics decreases the prevalence of the disease in broilers [60,61], while others have reported that such an effect is not significant [62]. In this study, there was no difference in footpad scoring for dermatitis between the two groups, while litter moisture was significantly lower at 24 days of age and comparable at 42 days. It must be also noted that, in general, the recorded scores were relatively low, indicating good management practices for the flock.

Probiotics are generally reported to increase antibody titers in poultry [63,64], although this is not always consistent [49,65]. The positive effects likely reflect a complex modulation of the immune system, rather than a simple enhancement of antibody production. Consequently, antibody titers do not always directly indicate the efficiency of the immune response. In this study, the lower antibody titer observed in probiotic-fed broilers 10 days after the second vaccination may reflect an early modulation of immunity, where probiotics support immune cell maturation and regulation in response to vaccination, rather than immediately boosting circulating antibodies as seen in the control animals. Over time, improved gut health, nutrient absorption, and the development of immunological memory likely contributed to a stronger and more sustained humoral response, resulting in higher antibody titers against NDV and IBV by day 42. Interestingly, in the case of IBD, where broilers were vaccinated only at the hatchery, the final antibody titer at day 42 was significantly increased in probiotic-fed birds, a result also reported by Rehman et al. [49]. This stronger effect is likely because the unvaccinated control birds had a relatively weak and inefficient baseline immune response, making the stimulatory effect of probiotics on antibody production more pronounced. Given that growth performance and feed conversion were significantly improved in probiotic-fed broilers and no signs of infection were observed despite challenges, these results suggest that the probiotic combination may promote a more efficient and durable immune response, favoring long-term protection rather than an early acute rise in antibodies.

The probiotic supplementation resulted in lower FABP2 concentrations at 24 days of age in relation to the control group. FABP2 (I-FABP) is a cytosolic protein released during enterocyte damage, widely validated as a circulating biomarker of intestinal epithelial injury and increased permeability in humans [66,67]. Although serum FABP2 has not yet been extensively characterized in poultry, its biological function is conserved, and FABP2 has been well studied in chicken intestinal physiology and gene expression [68,69]. Thus, lower FABP2 in probiotic-fed birds at 24 days is consistent with reduced epithelial stress at early stages of growth. FABP2 values in probiotic-fed birds at 42 days were comparable to those of the control group, indicating that late-phase elevations remained within the physiological range observed in non-supplemented birds. A plausible interpretation is that the probiotic combination exerts its influence primarily during the early developmental phase, when the intestine is more susceptible to epithelial disruption, but this effect does not extend into the finisher period. This notion agrees with previous work showing that probiotic effects on gut morphology and permeability tend to be strongest during early growth and can diminish as the gastrointestinal tract matures [70,71]. Although circulating FABP2 is not yet widely used in poultry research, its mechanistic relevance and established diagnostic value in mammals support its potential as an emerging biomarker for assessing enterocyte integrity in broilers. Combining serum FABP2 with established permeability markers (such as DAO, D-lactate, and endotoxin) and histological analysis would strengthen future evaluations of probiotic effects on gut barrier function.

## 5. Conclusions

The beneficial effects of probiotics are well documented in the relevant literature. In this study, the dietary supplementation of broilers with a probiotic combination based on Weizmannia faecalis (formerly Bacillus coagulans) and Bacillus licheniformis, under challenging rearing conditions, enhanced growth performance and increased the total tract digestibility of protein, fat and fiber, without affecting feed intake. In terms of carcass composition, the weight of the commercial parts was increased while carcass yield was not affected by the probiotic dietary supplementation. Finally, the antibody production against IBD at 42 days of age was increased and the FABP2 concentrations at 24 days of age decreased in the supplemented group in relation to the control group. Further research would be useful in terms of clarifying the appropriate dose as well as shedding light on the specific changes of the intestinal microbiota of broilers. Nevertheless, as the probiotic delivery systems continue to innovate parallel to the adoption of precision nutrition approaches, it is likely that the effectiveness and adoption of probiotics by the poultry industry will increase in the future.

## Figures and Tables

**Table 1:** Composition and calculated chemical analysis of experimental diets.

Ingredient	Starter (0–10 d)	Grower (11–24 d)	Finisher (25–42 d)
Corn	42.9	48.5	52.1
Wheat	10.0	10.0	10.0
Soymeal 47%	33.6	30.2	22.6
Sunflower meal 33.7%	4.5	4.0	4.0
Soy oil	3.3	4.2	4.8
Wheat bran	2.2	-	3.0
Soy protein 63%	-	-	1.0
Methionine DL	0.2	0.2	0.2
L-Lysine HCL	0.3	0.3	0.2
Choline 60%	0.1	0.1	0.1
Threonine	0.1	0.1	0.1
Salt	0.3	0.2	0.3
Vitamins and minerals premix ^1^	0.2	0.2	0.2
Monocalcium phosphate	1.1	0.9	0.6
Calcium	1.1	1.0	0.9
**Calculated Analysis (%)**			
Dry matter	87.9	87.9	87.8
Crude protein	22.9	21.0	19.0
Fiber	3.2	3.1	3.1
Fat	5.0	6.5	7.3
Ash	5.6	5.1	4.4
Calcium	0.96	0.88	0.76
Phosphorus total	0.67	0.59	0.51
Phosphorus avail.	0.48	0.44	0.38
Methionine	0.55	0.51	0.47
Digestible methionine	0.52	0.48	0.44
Lysine	1.43	1.30	1.15
Digestible lysine	1.25	1.14	1.00
ME (MJ/Kg)	12.5	12.8	13.4

^1^ Supplying per kg feed: 13,000 IU vitamin A, 4000 IU vitamin D3, 40 mg vitamin E, 9 mg vitamin K, 3 mg thiamin, 7 mg riboflavin, 6 mg pyridoxine, 0.035 mg vitamin B12, 40 mg niacin, 13 mg pantothenic acid, 1.5 mg folic acid, 0.13 biotin, 55 mg Zn, 155 mg Mn, 20 mg Fe, 12 mg Cu, 0.2 mg Co, 1 mg I, 0.2 mg Se, and phytase 0.2 g (2000 FTU/kg).

**Table 2:** Growth parameters and total tract digestibility of the experimental groups per feeding period.

Parameter ^1^	Control	Probiotics	SEM	*p*-Value
**DAY 0**				
BW (g)	45.8	45.3	0.4	0.503
**DAY 10**				
BW (g)	299.6	297.3	3.7	0.681
ADG (g/d)	25.4	25.2	0.4	0.719
CV (%)	9.5	9.8	0.6	0.295
FI (g/bird)	370.9	373.0	2.5	0.509
FCR (0–10 d)	1.242	1.256	0.061	0.629
**DAY 24**				
BW (g)	1105.8	1162.0	25.4	0.078
ADG (g/d)	57.6	61.8	1.6	0.490
CV (%)	9.3	8.9	0.9	0.802
FI (g/bird)	1260.1	1300.5	33.0	0.597
FCR (0–24 d)	1.583	1.514	0.051	0.283
**DAY 42**				
BW (g)	2575.4 ^b^	2822.7 ^a^	51.2	0.005
ADG (g/d)	81.6 ^b^	92.3 ^a^	2.9	0.049
CV (%)	9.5	8.7	1.0	0.743
FI (g/bird)	2808.7	2848.1	63.2	0.891
FCR (0–42 d)	1.735 ^a^	1.613 ^b^	0.032	0.022

^1^ BW = body weight, ADG = average daily gain, CV = coefficient of variation of body weights, FI = feed intake, FCR = feed conversion ratio. Means within a row with different superscripts differ significantly (*p* < 0.05).

**Table 3:** Nutrient total tract digestibility (%) of the experimental groups per feeding period.

Parameter	Control	Probiotics	SEM	*p*-Value
**DAY 10**				
Crude Protein	72.2 ^b^	76.1 ^a^	0.6	0.001
Fat Content	64.6 ^b^	71.9 ^a^	1.1	0.000
Fiber Content	43.9 ^b^	54.3 ^a^	0.6	0.000
**DAY 24**				
Crude Protein	68.6	70.1	0.7	0.097
Fat Content	76.0 ^b^	80.1 ^a^	1.1	0.029
Fiber Content	55.4 ^b^	59.4 ^a^	0.4	0.000
**DAY 42**				
Crude Protein	71.6	71.9	2.7	0.982
Fat Content	77.0	78.0	1.5	0.510
Fiber Content	64.8	67.1	2.8	0.380

Means within a row with different superscripts differ significantly (*p* < 0.05).

**Table 4:** Carcass parameters, footpad scoring, litter moisture and bone properties of experimental groups.

Parameter	Control	Probiotics	SEM	*p*-Value
Live weight (g)	2607.8 ^b^	2938.3 ^a^	60.6	0.001
Cold carcass (g)	1882.7 ^b^	2242.3 ^a^	43.1	0.000
Carcass yield (%)	72.3	76.7	2.3	0.278
**Bone properties**				
TT ^1^ weight (g)	19.2	20.2	0.4	0.063
TT length (mm)	105.5 ^b^	110.2 ^a^	0.8	0.000
Seedor index	182.5	182.6	3.5	0.982
**Footpad scoring**				
Day 24	0.5	0.4	0.1	0.879
Day 42	1.1	1.0	0.2	0.654
**Litter moisture**				
Day 24	26.1 ^a^	19.6 ^b^	1.0	0.000
Day 42	26.2	26.8	0.8	0.126
EPEF ^2^	344.9 ^b^	409.7 ^a^	14.0	0.007

^1^ TT = Tibiotarsus bone; ^2^ EPEF = European Poultry Efficiency Factor; Means within a row with different superscripts differ significantly (*p* < 0.05).

**Table 5:** Antibodies titer for NDV, IBV and IBD at 10, 24 and 42 days of age.

Parameter	Control	Probiotics	SEM	*p*-Value
**Newcastle Disease (NVD)**				
DAY 10	3675	3399	561	0.344
DAY 24	11,176	9113	633	0.099
DAY 42	7969	8277	568	0.828
**Infectious Bursal Disease (IBD)**				
DAY 10	2017	1599	355	0.678
DAY 24	186	241	58	0.772
DAY 42	8927 ^b^	9962 ^a^	238	0.026
**Infectious Bronchitis (IBV)**				
DAY 10	1022	858	308	0.883
DAY 24	387	211	142	0.172
DAY 42	1600	2016	235	0.485

Means within a row with different superscripts differ significantly (*p* < 0.05).

## Data Availability

The original contributions presented in this study are included in the article. Further inquiries can be directed to the corresponding author.
